# Oncolytic adenovirus-expressed RNA interference of O^6^-methylguanine DNA methyltransferase activity may enhance the antitumor effects of temozolomide

**DOI:** 10.3892/ol.2014.2442

**Published:** 2014-08-12

**Authors:** XIN-JUN CHEN, KAI ZHANG, YONG XIN, GUAN JIANG

**Affiliations:** 1 Clinical Laboratory, The Second Affiliated Hospital of Xuzhou Medical College, Xuzhou, Jiangsu 221002, P.R. China; 2 Department of Radiotherapy, Affiliated Hospital of Xuzhou Medical College, Xuzhou, Jiangsu 221002, P.R. China; 3 Jiangsu Key Laboratory of Biological Cancer Therapy, Xuzhou Medical College, Xuzhou, Jiangsu 221000, P.R. China

**Keywords:** cancer therapy, oncolytic adenovirouses, alkylating agents, RNA interference, short hairpin RNA, O^6^-methylguanine DNA methyltransferase

## Abstract

Temozolomide (TMZ) is an example of an alkylating agent, which are known to be effective anticancer drugs for the treatment of various solid tumors, including glioma and melanoma. TMZ acts predominantly through the mutagenic product O^6^-methylguanine, a cytotoxic DNA lesion. The DNA repair enzyme, O^6^-methylguanine DNA methyltransferase (MGMT), which functions in the resistance of cancers to TMZ, can repair this damage. RNA interference (RNAi) has been previously shown to be a potent tool for the knockdown of genes, and has potential for use in cancer treatment. Oncolytic adenoviruses not only have the ability to destroy cancer cells, but may also be possible vectors for the expression of therapeutic genes. We therefore hypothesized that the oncolytic virus-mediated RNAi of MGMT activity may enhance the antitumor effect of TMZ and provide a promising method for cancer therapy.

## Perspective

As a relatively recently identified alkylating (methylating) agent, temozolomide (TMZ) has become a focus of attention, most notably in malignant glioma and melanoma treatment (1,2). Resistance to TMZ occurs following prolonged treatment and therefore poses a major therapeutic challenge. A key mechanism of the resistance to TMZ is the overexpression of O^6^-methylguanine-DNA methyl transferase (MGMT) (3). MGMT repairs the TMZ-induced DNA lesion, O^6^MeG, by removing the methyl group from guanine to a cysteine residue (4). Suppressing MGMT activity, therefore, could enhance the cytotoxicity of TMZ against melanoma and glioblastoma multiforme (4).

In previous years, we have focused our research on oncolytic virotherapy. Oncolytic viruses exhibit selective replication and lysis in tumor cells, while also amplifying the expression and functions of therapeutic gene in the tumor microenvironment (5). Two main strategies are used for oncolytic adenovirus generation. One strategy is the deletion of the viral element that is required for replication of the virus in normal cells, but is dispensable in tumor cells, such as ONYX-015 or ZD55 with E1B-55K gene deletion (6,7). The other strategy is the use of a tumor-specific promoter to drive the gene that is required for viral replication (8). In clinical trials, the E1B 55-kDa-deleted oncolytic virus, ONYX-015, or the ONYX-015 derivative, H101, have exhibited encouraging anticancer activity when combined with chemotherapy (9).

RNA interference (RNAi) technology is able to downregulate targeted genes and has been evaluated as a potential therapeutic strategy in human cancer therapy (10). The knockdown of DNA repair genes by small interfering RNA (siRNA) and virally delivered short hairpin RNA (shRNA), can sensitize various cancer cells to chemotherapeutic agents *in vitro* (11). A previous study has shown that the use of siRNA to transiently transfect nasopharyngeal carcinoma cells and glioma cells results in the inhibition of MGMT gene expression and increased sensitivity to bis-chloroethylnitrosourea (12). Similarly, a study by Kato *et al* (13) revealed that the transduction of TMZ-resistant glioma cells with a LipoTrust™ liposome, which contains siRNA to inhibit MGMT gene expression, enhanced the sensitivity of the glioma cells to TMZ.

**Figure 1:**
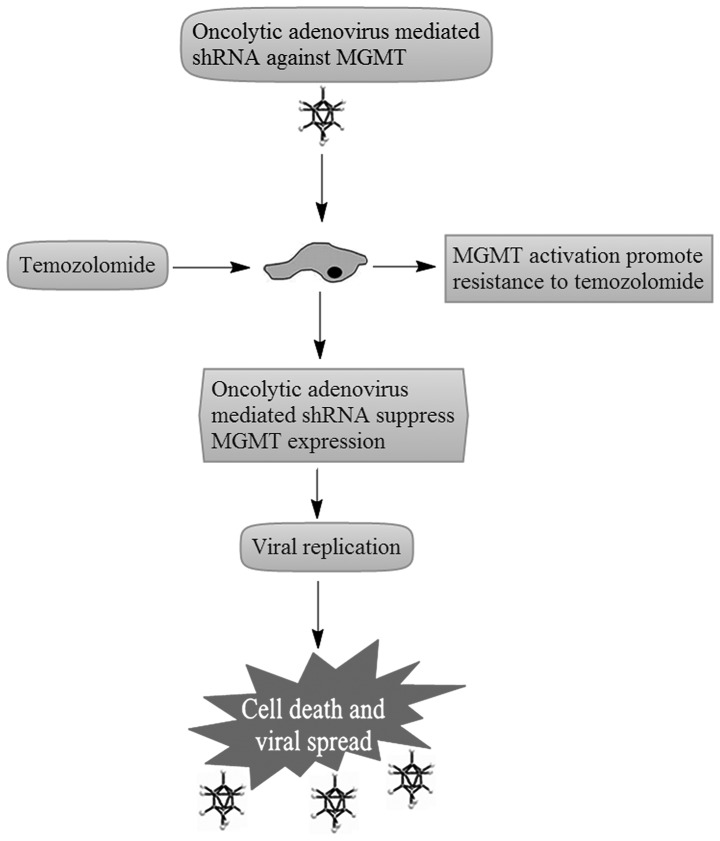
Schematic representation of MGMT downregulation by oncolytic adenovirus-armed shRNA to overcome temozolomide resistance in cancer cells. Following oncolytic adenovirus infection and replication, the inserted shRNA can target the DNA repair protein, MGMT, in tumor cells and multiply from several 100-fold to several 1,000-fold, in parallel with viral replication. The oncolytic adenovirus-armed shRNA targeting MGMT offers the advantage of an enhanced shRNA-mediated antitumor response through its intrinsic oncolytic activity. MGMT, O^6^-methylguanine DNA methyltransferase; shRNA, short hairpin RNA.

Zheng *et al* (14,15) focused on the production of several shRNA constructs using an oncolytic virus for delivery. Examples of these constructs included siRNAs against Ki67 and hTERT, which were observed to act as antiproliferative and apoptotic inducers in cancer cells. shRNA delivery via armed oncolytic viruses has potential for enhancing antitumor efficacy as a consequence of synergism between viral replication and oncolysis and shRNA antitumor responses (11). When conveying shRNA, oncolytic viruses are expected to effect a marked reduction in the tumor MGMT level, which should result in an increase in the cytotoxicity of TMZ (Fig. 1).

We hypothesize that the effects of the oncolytic virus-mediated RNAi of MGMT activity may enhance the cytotoxicity of TMZ in tumors for the following reasons: Firstly, the use of armed oncolytic viruses to deliver shRNA combines the advantages of gene therapy and virotherapy. The inserted shRNA can target the DNA repair protein, MGMT, in tumor cells and multiply by several 100- to several 1,000-fold in parallel with viral replication. The oncolytic adenovirus-armed shRNA targeting MGMT also offers the advantage of enhancing shRNA-mediated antitumor responses through its intrinsic oncolytic activity (10). Secondly, as a delivery agent that couples shRNA expression with viral replication, oncolytic adenoviruses can minimize the effects of off-target activity in normal cells, and facilitate, sustain and regenerate shRNA expression within the tumor microenvironment (15). Thirdly, as oncolytic adenovirus vectors and chemotherapeutic agents act by different mechanisms, there is a synergistic or additive effect rather than cross-resistance on the death of tumor cells (5).

The combination of these advantages and possibilities suggest that using oncolytic adenoviruses to deliver therapeutic shRNA targeting MGMT protein may be a powerful technique for overcoming resistance to TMZ in human cancers. This may result in a significantly enhanced antitumor outcome through MGMT-knockdown and viral oncolysis.
